# Combating Loneliness in Older Adults during the COVID-19 Pandemic: Findings from a Volunteer-Based Program in Greece

**DOI:** 10.3390/bs13100804

**Published:** 2023-09-27

**Authors:** Marianna Balta, Konstantinos Katsas, Chrysoula Grigoropoulou, Dimitrios V. Diamantis, Dimitrios Kalogiannis, Nikolaos Drougos, Eleni Fagogeni, Afroditi Veloudaki, Demosthenes Panagiotakos, Athena Linos

**Affiliations:** 1 PROLEPSIS Civil Law Non-Profit Organization of Preventive Environmental and Occupational Medicine, 7 Fragoklisias Street, 151 25 Marousi, Greece; m.balta@prolepsis.gr (M.B.); chrysoulg@gmail.com (C.G.); d.diamantis@prolepsis.gr (D.V.D.); d.kalogiannis@prolepsis.gr (D.K.); n.drougos@prolepsis.gr (N.D.); e.fagogeni@prolepsis.gr (E.F.); a.veloudaki@prolepsis.gr (A.V.); a.linos@prolepsis.gr (A.L.); 2 Medical School, National and Kapodistrian University of Athens, 115 27 Athens, Greece; 3 School of Health Science and Education, Harokopio University, 70 El. Venizelou Ave., 176 70 Athens, Greece; dbpanag@hua.gr

**Keywords:** older adults, social isolation, loneliness, COVID-19, helpline, pandemic, volunteering

## Abstract

The COVID-19 pandemic has exacerbated the feeling of loneliness, especially among older adults. This study aims to investigate any association between COVID-19 cases in Greece and the number of Loneliness Helpline calls at the Friendship at Every Age program and to assess whether the interconnection part of the program, which interconnects older adults with volunteers, can combat loneliness/social isolation in older adults. This is a supportive, volunteer-based, social intervention program. A total of 4033 calls were collected from July 2020 to November 2022, in Greece. Older adults who participated in the interconnection part completed baseline (*n* = 275) and follow-up questionnaires (*n* = 168), including the UCLA Loneliness Scale. A time-series analysis revealed a positive association in the number of calls with COVID-19 cases (Incidence Rate Ratio per 100 new COVID-19 cases = 1.012; Confidence Interval (95% CI) [1.002, 1.022]). A significant decrease in the Loneliness Scale was observed at follow-up [difference = −0.85; 95% CI (−1.16, −0.54)], with similar results by sex, educational level, and area of living. Loneliness Helpline calls increased during COVID-19 outbreaks, while the interconnection part had a positive impact on older adults, reducing their feeling of loneliness. Similar initiatives are required to better address the needs of the ageing population during and after health crises.

## 1. Introduction

The recent COVID-19 pandemic has caused a marked disruption of daily life globally and has especially burdened the vulnerable group of older adults. Specifically, in terms of physical health, older people are considered to be at higher risk of severe complications and mortality from COVID-19 and are disproportionately affected [1]. According to recent reports from the World Health Organization (WHO), the global pandemic has hampered the mental health of older people, degraded the care and support they receive, and led to a limitation of their social role [2,3]. Several studies confirm the significant impact of the unprecedented pandemic on mental health and, in particular, on the feeling of loneliness in older adults, which is closely linked to their mental health [2,4,5]. Such ramifications can further burden the ever-increasing ageing population worldwide and impose significant barriers to achieving healthy ageing and overall life satisfaction, particularly in the later stages of life [3,6,7].

Loneliness and social isolation are terms intricately interconnected and frequently utilized erroneously to denote one another, with the former referring to the subjective feeling of being alone or isolated and the latter to the lack of social contact or support [8]. To elucidate this ambiguity and highlight the impact of loneliness and social isolation on the individual, researchers have provided more coherent terminologies to differentiate the perceived feeling and the phenomenon. Thus, loneliness refers to the way people perceive and experience the lack of human interaction, while social isolation denotes an objective lack of meaningful and sustained communication [9].

While the establishment of sufficient social connections to uphold one’s social well-being may not pose a challenge for the majority of children and adults, the transition to older age can impose barriers to forming such relationships. The concurrent factors of dwindling economic resources, limitations in mobility, and mortality of contemporaries can establish a fertile environment for social isolation, ultimately setting the ground for detrimental consequences [7,10]. A considerable amount of data suggests that loneliness/social isolation has damaging effects on both physical and mental health in older adults, including a higher risk of anxiety, depression, cardiovascular disease, dementia, and all-cause mortality with the latter being a risk that might rival those of smoking, obesity and physical inactivity [10,11,12].

Evidently, the pandemic and the imposed social distancing measures have exacerbated the lack of social connectedness and have led to long-lasting consequences on the physical and mental health of older adults [13]. Even though the social isolation of older adults was put in the spotlight at the beginning of the pandemic [14], it quickly became evident that the act of frequent interactions could impose greater infection risks, leading to the rise of the COVID-19 Social Connectivity Paradox [15]. Within this paradox, most actions that could prevent social isolation, such as in-person social interactions, clashed with the social distancing measures, characterizing such interactions as a high risk of COVID-19 infection and leading to the promotion of further social isolation measures [15]. Consequently, older adults’ family members, friends, and loved ones followed the guidance of national public health bodies and kept their physical distance from older adults to avoid exposure of their loved ones to the virus [7,15]. Simultaneously, older adults, who were more altruistic than younger individuals, were more likely to agree with social distancing measures and therefore limit their social connections [16]. All these rapid and unprecedented limitations of social interactions have also led to the phenomenon of “lockdown loneliness”, referring to the exacerbation of disconnectedness during the monthly lockdowns, where almost all in-person social interactions became impossible [17]. Accounting for all these contributions to poor social isolation and loneliness, it is no wonder that this pandemic has been characterized as a “loneliness pandemic”, which is undeniably a public health concern [2,3,18].

This loneliness pandemic has been evident in Greece as well. In order to curb the unrestrained and rapid spreading of the virus and prevent an excessive burden on the nation’s healthcare system, Greece, like numerous other countries, adopted an array of measures, among which are the social distancing measures and stay-at-home directives. Leading up to the conclusion of 2021, Greece oscillated between “strict and stricter” measures, with the quarantine periods spanning several months and concluding in March 2021. Following what is commonly referred to as the second quarantine in March 2021 Greece had approximately 230,000 documented cases and 7180 COVID-19-related deaths [19]. Subsequently, Greece implemented a diverse range of strategies, designed to encourage vaccination uptake and curtail physical social interactions, particularly among unvaccinated individuals and high-mortality risk groups, such as older adults. Despite their initial effectiveness, following December 2021 a rapid increase in infection rate became apparent [19]. Nevertheless, the impact on Greek older adults loneliness was evident from the start, with more than one in five older adults reporting feeling more lonely a few months into the first quarantine [20] and with evident consequences on the quality of life [5], with loneliness persisting among older adults even two years later (Spring 2022) [21].

The need for support among older adults was deemed crucial and led to the creation of initiatives that attempted to empower them during the pandemic [22,23,24,25]. However, research on efforts that addressed the needs of older adults during that time, along with the fluctuations in the utilization of such programs through the pandemic, remains limited. In this article, the Friendship at Every Age program, a nationwide volunteer-based social intervention, is presented, with emphasis on its implementation during the COVID-19 era, in Greece. The aims of the present study were (a) to investigate whether there is an association between the number of daily COVID-19 cases in Greece and the number of calls received by the Loneliness Helpline of the Friendship at Every Age program and (b) to assess whether the implementation of the Interconnection part of the program (IC), can provide support against loneliness/social isolation of older adults.

## 2. Materials and Methods

### 2.1. Design

The Friendship at Every Age program is a supportive, volunteer-based social intervention initiative. However, in this study, the presented results will follow two study designs: (a) a prospective examination of the association between daily COVID-19 cases and the number of calls received by the Loneliness Helpline, and (b) a non-randomized non-controlled intervention study examining the outcome of the program to the beneficiaries’ perception of loneliness.

### 2.2. Setting

The Friendship at Every Age program is a comprehensive volunteer-based initiative, implemented by Prolepsis Institute, which is a civil law non-profit scientific organization. The program was established in July 2020 with the guidance of Les Petits Frères des Pauvres (PFP) [26]. Briefly, PFP is an international organisation based in France, with experience and extensive presence in supporting older adults since 1947. The organisation has established a large global network. Greece is one of the countries in which this initiative is implemented, with PFP executives providing expertise and guidance on training and implementation throughout the program.

The Friendship at Every Age program is free of charge, based on telephone communication only and has been active during the past three years continuously providing support to older adults. Through the years it was advertised nationwide in Greece, utilizing various communication channels, such as television and radio advertisements, leaflets, billboards throughout the cities and presentations at organizations or places with sufficient reach to older adults. Mouth-to-mouth was also crucial in informing older adults about the existence of the program.

### 2.3. The Two Main Pillars of the Friendship at Every Age Program

During the first three years of implementation, the program has specifically supported older adults and consists of two main pillars of action: (a) the Loneliness Helpline and (b) the interconnection part of the program (IC). As in the Loneliness Helpline, in the IC all volunteers were screened following a rigorous screening process and an online or in-person meeting. Following a short trial period, the volunteers who were considered acceptable for the purpose of the program (both the Helpline and IC) were assigned to one of the two main pillars.

#### 2.3.1. Loneliness Helpline

People over the age of 60 who suffer from social isolation and/or loneliness were invited to call the Loneliness Helpline. The aim of the Loneliness Helpline was to keep company to lonely older adults and to offer human contact. The Loneliness Helpline was operated by volunteers and was supervised by the program coordinators of the Prolepsis Institute. All volunteers participated in mandatory, uniform, and rigorous training, which explored the common potential physical and mental changes and needs of older adults and expanded on important communication skills to provide support to lonely older adults, such as active listening, empathy, providing clear and concise answers, handling disappointment/fear, exploring concerns, and effectively prompting questions. Other topics included depression and grief management, the effects of loneliness and social isolation on health, health promotion in older adults, but also COVID-19 issues relevant to older adults. All training was held live online and was made by the experts in each field (doctors, psychotherapists, social workers, etc.). The training of volunteers included practical sections too, with various scenarios and role-playing activities. Volunteers were instructed how and when to refer an older adult to another public service (helpline) when necessary and what to do in case of emergency. Their training included phone line operational issues too. After their training, volunteers were also provided with a document with communication guides and additional useful scenarios. Following this, there was a “trial” period of a month where volunteers would come to the institute where he/she would start the 3-h weekly shift of his/her choice and the coordinators were available in case there was any technical question regarding the phone line or any advice needed in relation to his/her communication with beneficiaries. Volunteers were supervised on a monthly basis by a certified psychiatrist and were supported by the program coordinators on a regular basis.

#### 2.3.2. Interconnection Part of the Program (IC)

At the end of a call to the Loneliness Helpline, each caller was asked whether he/she was interested in interconnecting with a trained volunteer for further, personalized social support. Those who agreed filled out a consent form with their personal information. The goal of the IC was the creation of new friendships through regular telephone communication (at least once per week) between the beneficiaries (older people) and their assigned trained volunteers. Volunteers were instructed to maintain a conversation lasting at least 30 min per session, unless the beneficiary wanted to end the conversation sooner, and to end the call when both parties agreed to do so. Following a short trial period, volunteers found out that calls lasted more than 30 min without presenting a burden to the beneficiaries. The training of the IC volunteers included a minimum of four hours of participation, with five main educational points of focus: active listening, depression and grief management, changes in lifestyle and needs of older adults, health promotion in older adults, and impact of COVID-19 in third age. All five points were accompanied by context-dependent scenarios and role-playing, with the first course being delivered by an expert in active listening and qualitative approaches, the second by a physiotherapist and a phycologist and the last three by a physician. During the calls, all volunteers aimed at maintaining a conversation in a friendly tone, discussing the beneficiaries’ difficulties, and providing supportive words and encouragement, with the goal of creating a sense of companionship. Even though the level of connection might not have reached the level of a traditional friendship due to the nature of the volunteer–beneficiary relationship, such interaction served as a valuable source of companionship and emotional support. Volunteers were advised to not stick to a pre-designed format for the calls, as the beneficiaries’ needs varied, but were encouraged to start the conversation by focusing on their concerns.

Beneficiaries who agreed to provide and share anonymously their data, completed a baseline questionnaire upon admission to the IC, which was conducted with telephone interviews, by the coordinators of the program. After 3 months of participating in the IC, follow-up structured questionnaires were completed. In total, 275 beneficiaries completed the baseline questionnaire, from whom 168 also completed the follow-up questionnaire (61% response rate).

### 2.4. Measuring the Connection of Daily Loneliness Helpline Calls and Daily COVID-19 Cases

#### 2.4.1. Number of Daily Loneliness Helpline Calls

The number of all successful calls from the date that the program was established (July 2020) up to November 2022, along with the date of the call, was recorded. This meant that during this timeframe volunteers kept a record of all successful calls (i.e., those where the volunteer explained the aim of the Helpline and engaged in conversation with the beneficiary) and the date of the call. This particular timeframe was chosen as the COVID-19 cases were routinely measured and the daily case count was high.

#### 2.4.2. Number of Daily COVID-19 Cases

To answer the first goal of the present work, i.e., the potential relationship between the number of COVID-19 cases with the number of calls received by the L.H., the reported daily COVID-19 confirmed cases were retrieved from the National Public Health Organization (NPHO) [27].

### 2.5. Measurements during the IC Participation

#### 2.5.1. Demographic Characteristics

During the first contact between the volunteer and the beneficiary of the IC, the volunteer requested the provision of the beneficiary’s basic sociodemographic characteristics. In particular, the following were collected: the participants’ age (in years), sex, and education level, classified into three groups, i.e., up to 6 years of school (low), 6 to 12 years (medium), and attended/graduated from higher education (high), marital status (not married, married, divorced, widowed), and area of residence were recorded.

#### 2.5.2. UCLA Loneliness Scale

A specific loneliness measurement tool was used, the revised three-item UCLA Loneliness Scale (UCLA-LS), which is a standardized, validated and widely used measure [28]. The UCLA-LS was used to quantify the impact of the Interconnection part of the program (b). It consists of 3 questions: “How often do you feel that you lack companionship?”, “How often do you feel left out?”, “How often do you feel isolated from others?”. Each question is rated on a 3-point scale (1 = Hardly Ever; 2 = Some of the Time; 3 = Often) and all items are summed to give a score between 3 and 9, with a higher score indicating a higher level of loneliness. Participants with scores 3–5 are classified as “not lonely” and 6–9 as “lonely” [28,29].

### 2.6. Bioethics

The Friendship at Every Age program was approved by the Ethical Committee of Prolepsis Institute (13844; N7-2021). The program was carried out in accordance with the Declaration of Helsinki. All participants in the Loneliness Helpline received adequate information about the program’s aim, design and implementation of the program, as soon as the call started. Written consent could not be obtained in the Helpline, as the calls were anonymous, but a clear explanation of the Helpline goals was given at the beginning of each call. Beneficiaries in the IC provided written informed consent to participate and share their data prior to participation. When written informed consent could not be attained due to social distancing measures, fear of infection or beneficiaries’ request to not provide exact details of their address, beneficiaries were asked to provide verbal consent based on the written informed consent structure, that was recorded with their permission. This study adhered to all relevant institutional regulations pertaining to the ethical utilization of human participants.

### 2.7. Statistical Analysis

#### 2.7.1. Evaluation of the Impact on Loneliness

Categorical variables are presented as relative frequencies (%) and continuous variables are presented as mean values (standard deviation). The association of UCLA-LS between baseline and follow-up was evaluated through Student’s paired t-test. Age classification into four groups was defined in accordance with quartiles. Sub-group analysis of UCLA-LS between baseline and follow-up was utilized by age, sex, educational level (low, medium, high), area of residence, cohabitation and marital status.

#### 2.7.2. Time Series Models

The number of calls is presented as daily counts. The number of calls was analysed by applying Negative Binomial regression analysis, due to overdispersion when Poisson regression analysis was applied. COVID-19 cases from 22 July 2020 to 6 November 2022 were added as an independent variable to investigate the association between the number of daily calls and COVID-19 confirmed cases. Additional explanatory variables were day-of-week (six “dummy” variables), holidays (binary variable indicating the dates that are official holidays) and every month from July 2020 to November 2022 (twenty-eight “dummy” variables). Goodness-of-fit was assessed by calculating the q-q plot of the standardized predicted residuals and the plot of predicted vs. observed values. The linear-trend model was estimated to evaluate differences in the number of calls during the studied period. Moreover, non-linear trends (i.e., parabolic, and fractional polynomial) were tested. Cross-correlation analysis was also applied between the time series of COVID-19 cases and the number of calls for five time periods that represented the main waves of the pandemic (July to December 2020, January to June 2021, July to December 2021, January to June 2022 and July to 6 November 2022). All tested hypotheses were two-sided. All statistical analyses were carried out using the Stata SE 16.1 software (STATA Corp Ltd., College Station, TX, USA).

## 3. Results

### 3.1. Number of Calls Received

The total number of calls during the studied period was 4033. Social isolation due to health issues was the most frequent reason for participating in the program (52%), while other important factors were living away from family/relatives/friends (40%) or the loss of relatives and friends (38%). More than 7 out of 10 participants were females (72%) and were living alone (79%), while more than half were widowed (56%).

### 3.2. Calls Received by the Loneliness Helpline and COVID-19 Cases

**Figure 1:**
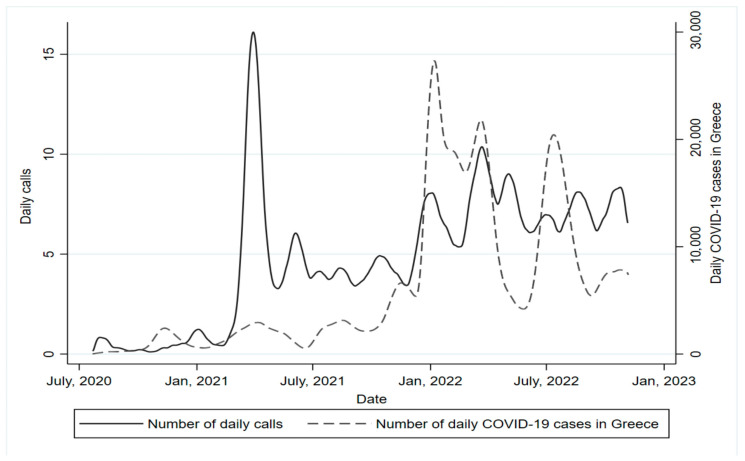
Daily number of calls and new COVID-19 cases in Greece, during 22 July 2020–6 November 2022 (lines derived from local polynomial regression). The daily number of COVID-19 cases was significantly associated with the number of calls in both the negative binomial model (*p* < 0.001) and cross-correlation analysis (all *p*’s < 0.05). Daily calls increased significantly between each year (*p* for linear trend <0.001).

From the date that the Friendship at Every Age program was established (i.e., July 2020), a significant increase in the number of daily calls was observed until June 2021 (*p* for linear trend <0.001); then, the number of daily calls regressed to the daily mean (i.e., 6 calls/day), but with an increasing trend up today (Figure 1). Time-series analysis revealed that the number of calls was significantly positively associated with COVID-19 cases, indicating that during the periods with the higher number of new COVID-19 cases, a higher number of calls would be observed, compared to days with less cases (IRR per 100 new COVID-19 cases = 1.012; Confidence Interval (95% CI) [1.002, 1.022]). Cross-correlation analysis confirmed the positive correlation between daily new confirmed COVID-19 cases and the number of calls. In particular, as the number of COVID-19 cases increased, the number of calls also increased from January to June 2021 (r = 0.615, lag 0 to 3 days, *p* < 0.001), July to December 2021 (r = 0.406, lag 0 to 3 days, *p* < 0.001), January to June 2022 (r = 0.318, lag 0 to 3 days, *p* < 0.001) and July to 6 November 2022 (r = 0.269, lag 0 to 7 days, *p* = 0.003) (Figure 1).

### 3.3. Significant Reduction in Loneliness—Interconnection Part

**Figure 2:**
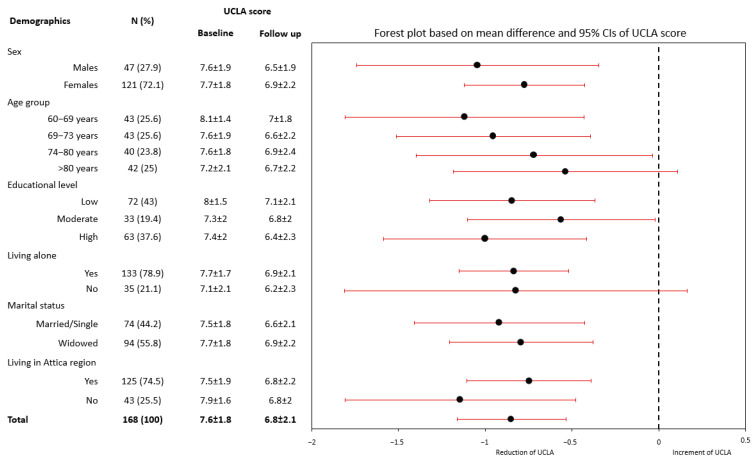
Difference in UCLA-LS score between follow-up and baseline, according to certain demographic characteristics (lower values indicate a higher reduction in Loneliness Scale). Abbreviations: UCLA Loneliness Scale (UCLA); 95% confidence interval (95% CI).

UCLA-LS difference is presented as a follow-up—baseline, with lower values indicating a higher reduction in loneliness. A significant decrease in the UCLA-LS was observed at follow-up, compared with baseline UCLA-LS (difference = −0.85 (2.0) units, *p* < 0.001) (Figure 2). Similar results were found when beneficiaries were categorized as “lonely” and “not lonely” (based on the UCLA Loneliness Scale score). Specifically, feeling “not lonely” was reported by 27.9% of the beneficiaries at follow-up, compared to 14.6% at baseline (*p* < 0.001). Changes of the UCLA-LS between follow-up and baseline were similar in both males and females, in all three educational level groups, among those living in the Athens metropolitan area and other cities, as well as by marital status (all *p*’s > 0.05). Moreover, age was inversely associated with changes in the UCLA Loneliness Scale score (rho = −0.139, *p* = 0.076; age groups between 60–69 years and 69–73 seem to benefit more from the IC) (Figure 2).

## 4. Discussion

The present study unveils that not only are Loneliness Helplines for older adults in higher demand during times of escalating COVID-19 cases, but also that such programs can be effective in mitigating loneliness. In particular, the study reports a substantial increase in the Loneliness Helpline calls of the Friendship at Every Age program during the COVID-19 outbreaks in Greece. This increase was more evident during the periods that COVID-19 cases increased and less profound when the epidemic curve tended to be flattened. Moreover, the Interconnection of older adults with volunteers proved to positively affect the feeling of loneliness after 3 months.

Interestingly, calls increased dramatically between February and June 2021, when COVID-19 cases had reached their greatest number by that time in Greece and restrictive measures for older adults were strictly applied, implying a greater need for support during that period [27]. This finding is in accordance with the increase of calls in similar helplines during restrictive measures [22,30]. At that point, it was evident that the impact of “lockdown loneliness” was amplified during monthly quarantines, and even though some older adults managed to find ways to regain social connectedness, those who experienced loneliness remained at a higher risk of experiencing poor mental health and depressive symptoms [17,31]. Efforts to address loneliness focused on leveraging the increasing interest in technology and smartphone usage among older adults. However, unequal access to digital resources and limited digital literacy posed barriers to reaching those who needed social connections the most [32,33,34]. As the contagion rate continued to rise and the detrimental effects of social isolation and fear of COVID-19 on mental health became more pronounced, actions aimed at reducing loneliness while providing safe connectedness and social support for social interaction might have become increasingly important for older adults [34,35,36]. Therefore, the availability of the Loneliness Helpline to older adults most probably played a crucial role in facilitating secure social connections during periods of unrest, such as periods of COVID-19 outbreaks.

Social isolation due to health issues was the most frequent reason for participating in this volunteer-based program and this is in agreement with evidence suggesting that multiple chronic conditions/poor health are risk factors for loneliness [37,38]. As expected, other factors leading to participation, such as living away from loved ones and the loss of relatives and friends highlight the well-documented value of significant others for older adults, especially during the pandemic, when social interaction was limited. Notably, it was found that during quarantine, family and friends were crucial comforting sources for older adults [39]. More than half of the participants seeking social support were widowed, a finding that comes in line with evidence that the prevalence rate of spousal bereavement increases with age, and this loss is associated with increases in loneliness over time [40]. The higher participation of females may be attributed to males being more reluctant in help-seeking/support, as supported by previous studies [41,42,43]. The vast majority of participants were feeling “lonely” independently of their socio-demographic profile. This suggests that loneliness can be present regardless of various social indicators, as also has been found in previous research, stressing the individuality of that experience [44]. Specifically, UCLA Loneliness Scale Score differences between baseline and follow-up were similar in most subgroups of older adults studied (gender, educational groups, area of residence, marital status), with lower scores achieved at follow-up, indicating the effectiveness of the Program. However, age was inversely associated with changes in the UCLA-LS score in our study, with the specific age groups of 60–69 and 69–73 years benefiting more from the IC. These observed differences in the level of benefit may be attributed to the slightly higher perceived loneliness of older adults aged less than 70 years at baseline. Such were the findings in a separate study conducted on older adults during COVID-19 social isolation measures, which indicated that older adults of younger age had lower odds of reporting loneliness compared to older adults of higher age [45]. Within our program, we theorize that younger older adults, especially those who experienced a more pronounced impact of the pandemic on their social connections, had fewer means to communicate with others, resulting in a heightened sense of loneliness and were more likely to reach out to the helpline. In contrast, older age groups less proficient with technology were less inclined to explore alternative communication methods and thus greater variance in their loneliness could be observed. Additionally, the significant diversity within our group of older adults might contribute to this subtle discrepancy. Nonetheless, the results showed a significant reduction in loneliness among all other groups, as aforementioned, illuminating the probable effectiveness of such initiatives.

The COVID-19 pandemic made more salient a “loneliness pandemic” among older adults, by exacerbating the feelings of loneliness/social isolation, since social interactions were reduced [5]. This “loneliness pandemic” unarguably constitutes an alarming public health concern [18]. In an effort to address this, several initiatives were implemented globally. For instance, the Age-Friendly Student Senior Connection program was designed to interconnect graduate students with isolated older adults to decrease social isolation, during COVID-19, in California. Students were thoroughly trained and provided 30 to 60-min phone calls to community-dwelling older adults, multiple times per week, for 6 weeks [23]. Similarly, in Chicago, healthcare professional students offered psychosocial support through telephone to older adults living in long-term care facilities and the community, through the Seniors Overcoming Social Isolation (SOS), an outreach program [24]. In Greece, a telephone Helpline was established to promote psychosocial wellbeing during COVID-19, including loneliness, which was reported as a major challenge for most of the calls [22]. Findings from a recent review highlight that such programs can lower the feeling of loneliness in older adults or reduce its negative outcomes [25]. These further complement the importance of the Friendship at Every Age program for older adults in Greece. Furthermore, these findings shed light on the need for similar comprehensive initiatives globally, especially during public health crises and their aftermath. Nonetheless, the adaptable nature of such programs, renders them applicable in a myriad of emergencies or circumstances wherein lonely older adults encounter challenges in being reached through conventional means. Such scenarios encompass providing comfort and company to older adults in conflict-ridden regions, in remote areas or in areas with limited access and across areas profoundly affected by natural disasters still interconnected by communication networks. Additional applications of such programs include reaching out to older adults whose illness mandates social isolation or those with physical impairments precluding physical interaction outside of their houses.

As with all studies, this study has some limitations. This study is limited by its non-controlled design, as it was not possible to include a control group, due to ethical concerns. Such non-profit initiatives are often bound by this limitation, as they distribute all of the budget to the intervention provision. The sample size (*n* = 168) was small and consisted only of beneficiaries from a single country, Greece. However, to the best of the authors’ knowledge, this is the largest program aiming to combat the loneliness of older people through both the provision of a Loneliness Helpline and regular communication with assigned trained volunteers (IC) that has been implemented in Greece. Additionally, most of the sample (74.5%) was concentrated in the capital and thus, older adults living in rural areas were underrepresented. Nonetheless, the Institute aimed at making the program widely available to all people living in Greece aged over 60 years, with a simple application process. Regarding the questionnaires, loneliness measurement constitutes a challenge since it is a subjective experience. However, the UCLA Loneliness Scale used in questionnaires is a standardized, validated and widely used tool. Regarding the calls, there is no feedback on caller satisfaction, because the primary objective of the Loneliness Helpline was the provision of anonymous support. Lastly, data are available for only a short period of time and thus, further studies are required for the assessment of the long-term effect of this program on the loneliness of older adults.

## 5. Conclusions

During the COVID-19 era, the Friendship at Every Age program provided an effective method of combating the loneliness of older adults. A response in the form of a Loneliness Helpline and aiming at the creation of new friendships through regular telephone communication with assigned and trained volunteers (IC), is potentially an effective initiative to support older adults. Loneliness Helplines for older adults during periods of public unrest such as COVID-19 outbreaks can often be crucial in eliminating the negative consequences of social isolation and loneliness. Our findings can be used to raise awareness among the public and the state, as additional similar initiatives are required in order to better address the needs of the ageing population during and after health crises.

## Data Availability

The data presented in this study are available on request from the corresponding author.
